# Protocadherin-1 Localization and Cell-Adhesion Function in Airway Epithelial Cells in Asthma

**DOI:** 10.1371/journal.pone.0163967

**Published:** 2016-10-04

**Authors:** Grissel Faura Tellez, Brigitte W. M. Willemse, Uilke Brouwer, Susan Nijboer-Brinksma, Karl Vandepoele, Jacobien A. Noordhoek, Irene Heijink, Maaike de Vries, Natalie P. Smithers, Dirkje S. Postma, Wim Timens, Laura Wiffen, Frans van Roy, John W. Holloway, Peter M. Lackie, Martijn C. Nawijn, Gerard H. Koppelman

**Affiliations:** 1 Department of Pediatric Pulmonology and Pediatric Allergology, Beatrix Children’s Hospital, University of Groningen, University Medical Center Groningen, Groningen, The Netherlands; 2 Department of Pathology & Medical Biology, Experimental Pulmonology and Inflammation Research, University of Groningen, University Medical Center Groningen, Groningen, The Netherlands; 3 GRIAC Research Institute, University of Groningen, University Medical Center Groningen, Groningen, The Netherlands; 4 Department of Biomedical Molecular Biology, Ghent University & Inflammation Research Center, VIB, Ghent, Belgium; 5 Laboratory for Molecular Diagnostics - Hematology, Ghent University Hospital, Ghent, Belgium; 6 Clinical & Experimental Sciences, Faculty of Medicine, University of Southampton, Southampton, United Kingdom; 7 Human Genetics and Genomic Medicine, Human Development & Health, Faculty of Medicine, University of Southampton, Southampton, United Kingdom; 8 Brooke Laboratory, Academic Unit of Clinical and Experimental Sciences, Faculty of Medicine, University Hospital Southampton, University of Southampton, Southampton, United Kingdom; 9 Department of Pulmonology, University of Groningen, University Medical Center Groningen, Groningen, The Netherlands; 10 Department of Pathology & Medical Biology, University of Groningen, University Medical Center Groningen, Groningen, The Netherlands; Emory University School of Medicine, UNITED STATES

## Abstract

**Background:**

The asthma gene *PCDH1* encodes Protocadherin-1, a putative adhesion molecule of unknown function expressed in the airway epithelium. Here, we characterize the localization, differential expression, homotypic adhesion specificity and function of PCDH1 in airway epithelial cells in asthma.

**Methods:**

We performed confocal fluorescence microscopy to determine subcellular localization of PCDH1 in 16HBE cells and primary bronchial epithelial cells (PBECs) grown at air-liquid interface. Next, to compare PCDH1 expression and localization in asthma and controls we performed qRT-PCR and fluorescence microscopy in PBECs and immunohistochemistry on airway wall biopsies. We examined homotypic adhesion specificity of HEK293T clones overexpressing fluorescently tagged-PCDH1 isoforms. Finally, to evaluate the role for PCDH1 in epithelial barrier formation and repair, we performed siRNA knockdown-studies and measured epithelial resistance.

**Results:**

PCDH1 localized to the cell membrane at cell-cell contact sites, baso-lateral to adherens junctions, with increasing expression during epithelial differentiation. No differences in gene expression or localization of PCDH1 isoforms expressing the extracellular domain were observed in either PBECs or airway wall biopsies between asthma patients and controls. Overexpression of PCDH1 mediated homotypic interaction, whereas downregulation of PCDH1 reduced epithelial barrier formation, and impaired repair after wounding.

**Conclusions:**

In conclusion, PCDH1 is localized to the cell membrane of bronchial epithelial cells baso-lateral to the adherens junction. Expression of PCDH1 is not reduced nor delocalized in asthma even though PCDH1 contributes to homotypic adhesion, epithelial barrier formation and repair.

## Introduction

In 2009, our group identified *Protocadherin 1* (*PCDH1)* as a susceptibility gene for bronchial hyperresponsiveness (BHR) and asthma [1]. Subsequent studies in Dutch [2], German [3], and Danish populations [4] reported associations of different *PCDH1* gene variants with multiple phenotypes of asthma as well as eczema. Since different *PCDH1* gene variants were associated with specific asthma phenotypes including BHR positive [1], early onset [4], and non-allergic asthma [3], we proposed that PCDH1 may contribute to disease pathogenesis in subgroups of asthma patients [5].

Recently, we detected strong expression of PCDH1 in the airway epithelium [1]. We found two different *PCDH1* mRNA transcripts encoding, respectively, protein isoform-1 (150 kD) and isoform-2 (170 kD) [1,6] which share the extracellular and transmembrane domains, but differ in their intracellular domains. While isoform 1 of PCDH1 has a relatively short intracellular tail lacking conserved domains (CMs), the longer PCDH1 isoform 2 encodes an additional intracellular domain with three CMs likely involved in signal transduction. In a subsequent study, we identified a shorter third isoform which lacks the extracellular domain yet contains the intracellular tail and hypothesized that this isoform 3 may also act as a signaling molecule. Noteworthy, we showed that expression levels of PCDH1 mRNA and protein isoforms increased during differentiation of primary bronchial epithelial cells (PBECs) cultured under air-liquid interface (ALI) conditions [6], indicating that PCDH1 might contribute to bronchial epithelial cell differentiation or establishment of the epithelial barrier, a process that is impaired in asthma [7]. The reduced barrier function and damaged phenotype of the airway epithelium is thought to contribute to pathogenesis of asthma [8]. Recently, *Kozu et al*. found that siRNA silencing of PCDH1 increased epithelial permeability in 16HBE cells suggesting that PCDH1 is important for the physical barrier of the airway epithelium [9].

At present, more detailed information confirming the specific subcellular localization of PCDH1 in the airway epithelium is lacking and its contribution to asthma susceptibility is not fully understood. As PCDH1 may act both as a cell-cell adhesion molecule [9] and a signaling molecule, it is relevant to characterize the subcellular localization of PCDH1 in airway epithelial cells, its adhesive specificity and evaluate its contribution to epithelial barrier function during repair after wounding. Moreover, it is not known whether expression or localization of PCDH1 is altered in the airway epithelium of subjects with asthma compared to healthy controls. We hypothesized that PCDH1 is required for effective epithelial barrier formation and repair, and that expression is reduced in asthma [6]. To test our hypothesis, we characterized expression levels and localization of PCDH1 in ALI cultured PBECs and in airway wall biopsies from asthma patients and control subjects. Furthermore, we investigated if cells expressing PCDH1 showed evidence for homotypic adhesion and assessed the functional consequences of PCDH1 downregulation for airway epithelial barrier function and epithelial repair.

## Material and Methods

### Plasmid construction

Human PCDH1 cDNA isoform 1 (pCMV-XL4-iso1) and 2 (pCMV-XL6-iso2) plasmids (OriGene, Rockville, USA) were used as template for PCR using specific primers carrying *Hind*III and *Not*I restriction sites. PCR was performed with Phusion^™^ High Fidelity (F553L, Finnzymes, Fisher Scientific, Landsmeer, The Netherlands). PCR products were incubated with *Hind*III and *Not*I endonucleases (New England Biolabs, Frankfurt, Germany) and subcloned in optimized pcDNA3.1eGFP (kindly provided by Robert Tsien, UCSD) and used for transient transfection). Orientation and cDNA sequence of the resulting expression plasmids were confirmed by DNA sequencing.

### Overexpression of PCDH1 isoforms 1 and 2

16HBE cells were seeded on 12mm glass coverslips (Thermo Scientific Menzel, Landsmeer, The Netherlands) coated with 30 μg/ml collagen and 10 μg/ml BSA in EMEM. After 24h cells were transfected with 1 μg of expression plasmids encoding either the PCDH1-GFP fusion proteins or GFP (green fluorescent protein) using Lipofectamine^®^-2000-reagent (Invitrogen Life Technologies, Bleiswijk, Netherlands) according to the manufacturer’s instructions. At 80–100% confluence, cells were fixed in 3.5% formalin for 15 min and washed with 1x PBS at room temperature. Localization of the PCDH1 fusion proteins was monitored by confocal microscopy.

### Plasmids and cell aggregation assay

Clones of HEK293T cells stably expressing human PCDH1-isoform-1-GFP were generated as described previously [10]. The control vector expressing EGFP was made by digesting the pLenti6-EGFP destination vector with EcoRV, which removes the Gateway cassette. The vector backbone was then religated to itself to obtain an EGFP-expressing construct. The pLenti-PCDH1-isoform-2-EYFP vector was made by first performing an LR reaction between the PCDH1-isoform-2 entry vector and the pdEYFP vector, resulting in the pdEYFP-PCDH1-isoform-2 plasmid. This was then digested with NotI, blunted with Pfu polymerase and digested with NheI. The resulting fragment was introduced into the pLenti6-PCDH1-isoform-2 vector, which had been digested with Csp45I, blunted with Pfu polymerase and digested with NheI. The PCDH1-isoform-2 insert was removed by performing a BP reaction with pDONR207 to obtain the pLenti6-EYFP destination vector. The Gateway cassette was largely removed by digesting the latter plasmid with EcoRI and allowing the plasmid to self-ligate. This resulted in a pLenti6 vector expressing only EYFP. HEK293T cells were cultured in DMEM containing 10% fetal calf serum. For the production of lentiviral particles, HEK293T cells were transfected with the appropriate pLenti6 plasmid (Invitrogen) together with pMD.G and pCMV Delta R8.9 (obtained from Dr. Didier Trono, School of Life Sciences, Lausanne, Switzerland). After 48 h, supernatants were filtered through a 0.45 μm filter (Millipore) and added to the target cells. 24 h after transduction, cells were transferred to a new culture vessel and antibiotics were added 24 h later.

Cell aggregation assays were performed by mixing two different combinations of HEK293T clones with a ratio of 1:1 to give a final concentration of 3×10^5^ cells/ml, followed by rotation for 4 hours at 37°C. Mixed clones were seeded in 24-well plates on top of coated 12 mm glass coverslips for better imaging and incubated for 20h at 37°C. Aggregates were then fixed in 3.5% formaldehyde solution for 20 min and washed with 3x PBS at room temperature. Fluorescence detection was observed using a Zeiss LSM 780 two photon/single photon confocal laser scan microscope.

Confocal images were scored by three individual observers, using a grid with 9 random points to identify a reference cell. This cell expressed either EGFP or YFP. The observers scored the fraction of cells in contact with the reference cell expressing either the same fluorescent marker as the reference cell or the other fluorescent marker compared to the reference cell. Thus, every independent experiment yielded 9 observations of the fraction of cells in contact to the reference cell either expression the same fluorescent marker of the other. The observer was blinded to the clone’s mixtures throughout the image analysis.

### Cell culture and Primary Bronchial Epithelial cells (PBECs)

16HBE 14o- (16HBE) cells, an immortalized normal bronchial epithelial cell line [11] were donated by Dr D.C Gruenert (Department of Medicine, University of Vermont, and University of California, SF, USA). 16HBE cells were cultured in Eagle minimum essential medium (EMEM) (Biowhittaker, Verviers, Belgium) containing 10% FCS (FCS; Hyclone, Logan, UT) supplemented with 100 U/ml penicillin and 100 μg/ml streptomycin as described previously [12]. HEK293T-PCDH1 clones were cultured in Dulbecco's Modified Eagle's medium (DMEM) supplemented with 10% fetal calf serum (FCS; Biowhittaker, Verviers, Belgium) and puromycin (0.5μg/ml; A11138-03, Gibco^®^/Life Technologies, Bleiswijk¸ The Netherlands) in T25-flasks.

#### A. Bronchial brushings from University Medical Center Groningen

Tracheobronchial tissues were obtained according to standard guidelines from left-over tracheobronchial tissue of donor lungs, of whom no further information was available and treated with pronase, as described previously [13]. Primary bronchial epithelial cell (PBECs) were cultured on coated flasks with 30 μg/ml collagen, 30 μg/ml fibronectin, and 10 μg/ml BSA in serum-free hormonally supplemented bronchial epithelium growth medium BEGM (Clonetics, LONZA, Breda, The Netherlands) and passaged twice. PBECs were seeded in coated permeable polyester membrane (0.4 μm) (Corning-Costar, Corning, NY). After reaching confluence, cells were exposed to air and cultured under ALI conditions as described previously [12]. Mucociliary differentiation was confirmed between days 14 and 28 after exposure to ALI by transepithelial electrical resistance (TEER) measurement and by presence of mucus and cilia. The study protocol was consistent with the Research Code of the University Medical Center Groningen (http://www.rug.nl/umcg/onderzoek/researchcode/index) and national ethical and professional guidelines (“Code of conduct; Dutch federation of biomedical scientific societies”; http://www.federa.org).

#### B. Bronchial brushings from University Hospital Southampton

Bronchial brushings were obtained by fiber-optic bronchoscope [14] according to standard procedures from subjects without or with asthma and grown under ALI conditions [15]. These procedures were ethically approved by the Southampton and South West Hampshire Research Ethics Committees (REC no. 05/Q1702/165, 13/SC/0182 and 09/H0504/109) including written informed consent received from all volunteers. Asthma was diagnosed by physicians according to the British Thoracic Society guidelines. Subjects without asthma were healthy subjects selected from the University of Southampton volunteer database. They had no history of asthma and no underlying disease. Details of the study subjects are described in Table 1. Different subsets of patients were used to study differences in either PCDH1 localization or gene expression as sample size was limited by the number of PBECs available.

**Table 1:** Clinical characteristics of asthma and control PBECs cultured in ALI.

Characteristics	PBECs	
	Asthmatics†	Control
Subjects (number)	10	11
Sex (male/female)	3/7	5/6
Age (years) (median, range)	37.5 (24–66)	38 (21–70)
FEV1 (% predicted) (median, range)	88 (33.2–112)	102 (83–134.9)
Smoking (no/current/ex)	9/0/1	8/1/2
Inhaled corticosteroids	10	0

^†^BHR to methacholine was not performed.

### Immunofluorescence staining (IF)

#### A. PCDH1 in 16HBE human bronchial epithelial cells

16HBE cells were seeded on 12 mm glass coverslips coated as described above. After 24h, cells were transfected with 50 pmoles of human PCDH1-siRNA targeting Exon 2 (SASI_Hs01_00051867; Sigma-Aldrich, Zwijndrecht, The Netherlands) or universal negative control-siRNA (SIC001-10nmol, Sigma-Aldrich, Netherlands) with Lipofectamine^®^ RNAiMAX (13778–075, Invitrogen Life Technologies, Netherlands). 96 h after transfection, cells were fixed with ice-cold acetone:methanol (1:1) for 20 min, followed by PBS washes.

Next, cells were blocked in 10% normal goat serum in 1% BSA in PBS for 20 min and incubated for 1 h with either 1% BSA in PBS (Negative control) and a mouse monoclonal antibody PCDH1-EC1, directed towards the N-terminal EC1 domain of human PCDH1 (H00005097-M01; Abnova, Taipei, Taiwan) diluted in 1% BSA in PBS; followed by an Alexa Fluor^®^ 594 conjugated secondary antibody (Life Technologies Ltd, Bleiswijk, Netherlands) for 1 h at room temperature. Negative controls were obtained by replacing the primary antibody with 1% BSA in PBS. All samples were stained with DAPI (D9542-1MG, Sigma-Aldrich, Zwijndrecht, The Netherlands) diluted 1:50, and mounted in antifade reagent (Citifluor Ltd., London, United Kingdom). Fluorescence staining was detected using a confocal microscope Leica SP8.

#### B. PCDH1 localization during differentiation in PBECs cultured under ALI conditions (IF)

PBECs from lung donors were grown under ALI conditions at the University Medical Center Groningen and sacrificed at five time points: 0 (start of air exposure), 7, 14, 21, and 28 days after air exposure. Transwell inserts were washed in PBS supplemented with 0.01% CaCl_2_ and fixed in acetone:methanol for 5 min. Inserts were subjected to immunofluorescence staining and confocal microscopy as described above.

#### C. Double staining (IF) in differentiated PBECs cultured under ALI conditions

PBECs grown for 28 days under ALI conditions at the University Hospital Southampton and fixed and stained as described above. For double staining, cells were incubated for 1 h with either 1% BSA in PBS (negative control) or with primary antibodies also diluted in 1% BSA in PBS. Sequential immunostaining was as follows: PCDH1-EC1 (H00005097-M01) and Alexa Fluor^®^ 594 conjugated secondary antibody; and later with antibodies directed to E-cadherin (H-108; Santa Cruz Biotechnology, INC, Heidelberg, Germany), Occludin (18–7431; Invitrogen Life Technologies Ltd, Bleiswijk, Netherlands), or β-Tubulin IV (T7941, Sigma-Aldrich, Zwijndrecht, The Netherlands), followed by an Alexa Fluor^®^ 488 conjugated secondary antibody (Life Technologies Ltd,). Finally, all samples were treated as mentioned above and studied by confocal microscopy.

#### D. PCDH1 in asthma vs. control PBECs cultured under ALI conditions

PBECs derived from healthy and asthmatic patients (n = 4 per group) grown under ALI conditions for a minimum of 21 days at the University Hospital Southampton were fixed and stained with monoclonal antibody PCDH1-EC1 (H00005097-M01) in combination with the Zenon^®^ Alexa Fluor^®^ 647 Mouse IgG_1_ Labelling Kit (Z-25008, Life Technologies Ltd, Bleiswijk, Netherlands) according to the manufacturer’s instructions.

Fluorescence images were acquired with a 1.4/oil NA, 63X objective on a Leica SP8, Confocal Microscopy (Leica Microsystems B.V, Rijswijk, Netherlands). Imaging of DAPI, Alexa Fluor 488, Alexa Fluor 594 and Alexa Fluor 647 fluorescence was performed by excitation using 405, 488, 552 and 638 nm laser lines, respectively. For comparison within each experiment laser intensity and pinhole remained the same whereas optimal gain and offset settings were chosen specifically for each condition.

### qRT-PCR

Healthy and asthmatics PBECs cultured at ALI condition for a minimum of 21 days were subjected to RNA extraction using TRIzol reagent (Invitrogen, Paisley, UK) following manufacturer’s instructions. Contaminating gDNA was enzymatically removed (DNA-free kit, Ambion, Austin, USA). RNA template was converted to cDNA with the NanoScript reverse transcription kit (PrimerDesign Ltd, Southampton, UK).

Quantitative RT-PCR experiments were performed using 20 ng of cDNA and 5 μl TaqMan Gene expression master mix (Applied Biosystems^®^, Life Technologies, Bleiswijk, The Netherlands) as previously described [6]. Gene expression was assessed for: PCDH1 (assay exon 1–2 exon, Hs00170174_m1; assay exon 3–4 exon, Hs00260937_m1; Life Technologies, Bleiswijk, The Netherlands), and a pre-designed assay for E-cadherin/CDH1 (Hs01023894_m1, Life Technologies, Bleiswijk, The Netherlands) as a house-keeping gene. qRT-PCRs were measured in a 384-well format on the ABI7900HT cycler. All experiments were performed in duplicates and analyzed using SDS 2.1 Software. Ct (cycle threshold) values were averaged and normalized to E-cadherin.

### Tissue samples

Airway biopsies from asthma patients and matched controls were obtained by fibre-optic bronchoscopy for immunohistochemical analyses. Tissues were fixed in ice-cold acetone in the presence of protease inhibitors iodoacetamide (20 mM) and phenylmethylsulphonylfluoride (PMSF; 2 mM), stored at -20°C for 24 h and then processed into water-soluble glycol methacrylate resin [16]. This study was approved by the Local Research Ethics Committee (06/Q1702/109) and the University Hospital Southampton NHS Foundation Trust Research and Development Committee (RHMCH10395). Written informed consent was obtained. Details of the study subjects are described in Table 2.

**Table 2:** Clinical characteristics of subjects used to study differences in PCDH1 expression using immunohistochemistry on human airway wall biopsies.

Characteristics	Control Subjects	Mild Asthmatics	Severe Asthmatics†
Subjects (number)	8	10	10
Sex (male/female)	6/2	3/7	4/6
Age (years) *	25 (21–38)	20 (18–24)	56 (35–63)
FEV1% of predicted pre b’dil *	100 (85.3–113)	97.7 (82.8–129.6)	46.2 (26.5–65)
FEV1% of predicted post b’dil*	104.8 (89.1–117)	104.4 (91.7–133)	57.5 (29.2–68.5)
Reversibility (%) *	4.5 (0.0–10.0)	7.2 (2.6–16.1)	28.3(0.0–56.0)
PD20 (mg/ml) to methacholine	>16	2.522 (0.12–9.11)	N.A
Atopy (Yes/No/unknown)	1/6/1	10/0/0	5/5/0
Inhaled Corticosteroids (mcg/day)	0	0	2000 (800–3600)
Oral Corticosteroids (mg/d)	0	0	12
Asthma Control Questionnaire *	0	0.34 (0.0–0.83)	3.3 (0.8–4.86)
Smoking History (Current/ex/No)	0/1/7	0/0/10	2/2/6

N.A = not available; Pre b’dil, = pre bronchodilator; Post b’dil = post bronchodilator.

*Values are presented as mean (range);

^†^BHR to methacholine was not performed in the severe asthmatics.

### Immunohistochemistry –IHC

Glycolmethacrylate (GMA) tissue sections of approximately 2μm were cut using a Leica Supercut RM 2065 Microtome and pre-treated with a solution of 0.1% sodium azide and 0.3% hydrogen peroxide for 30 min to inhibit endogenous peroxidase and then blocked with culture medium for 30 min. Then, sections were incubated overnight with either mouse monoclonal PCDH1-EC1 (H00005097-M01), diluted 1:10, or with TBS (negative control). Following a washing step and a two-hour incubation with biotinylated secondary rabbit anti-mouse Ig antibody (dilution 1:1200), a further washing step and a 2-h incubation with avidin biotin-peroxidase complex (1:75) were done. Immunoreactivity was revealed using diaminobenzidene and counter-stained with Mayer's haematoxylin. Staining intensity was assessed using a computerized assessment with the Fiji distribution of Image J [17]. Images were acquired using a 40X objective on a Nikon Eclipse E600 Photomicroscope (Nikon UK Limited, Surrey, United Kingdom). For each slide, the cross-sectional area of the airway epithelium was assessed based on the pre-defined inclusion criteria (see below). Image analysis consisted of interactively segmenting the immunostained area for each slide to calculate the percentage of the epithelial area stained. Each slide contained 2 stained sections and providing the epithelium met the inclusion criteria an average percentage was calculated. This was repeated twice on different days and the mean of the three percentages calculated. The observer was blinded to the sample classification throughout the image analysis. Inclusion criteria to select epithelium for Image J analysis were defined as follows: the epithelium was present from basement membrane up to the apical surface of the columnar cells and not damaged or tangentially cut; the epithelium was attached to underlying tissue and finally, there was no or minimal background (non-specific) staining.

### Measurement of epithelial barrier function

To assess epithelial barrier function we measured electrical resistance and capacitance of a confluent monolayer of 16HBE cells using the ECIS system (Electrical Cell substrate Impedance Sensing) as described [12]. First, expression of PCDH1 was downregulated in 16HBE cells by transfection of either 30 or 50 pmoles PCDH1-siRNA. Positive control protein E-cadherin was downregulated by E-cadherin-siRNA (directed against sequence GCAGAAUUGCUCACAUUUC) (773797 & 773798, Eurogentec, Maastricht, The Netherlands) and universal negative control-siRNA was used as a negative control, as described above. After 24 h, transfected cells were reseeded at 10^5^ cells/well on collagen-coated 8-well arrays model 8W10E (Applied Biophysics, Troy, NY, USA) and resistance was measured for 150 h.

A wounding experiment was also performed using a delayed transfection approach whereas 16HBE cells were directly seeded on collagen-coated 8-well arrays at 5×10^4^ cells/well and allowed to attach for 24 h before transfection with respective siRNAs as previously described. Here, 90 h after seeding the transfected cells, the confluent layer was wounded by electroporation using voltage pulses of 5 V and 40 kHz for 30s and the subsequent morphological wound healing response was monitored by measuring restoration of electrical resistance for another 17 h, as well as the capacitance. Electrical resistance values for all conditions were normalized to those of the negative control-siRNA condition at the latest measurement prior to wounding (which was set to 100%). Different designs were used to obtain specific timing of PCDH1 knockdown: either initially during build up of resistance, or during repair after wounding. The frequency for studying electrical resistance was set at 400 Hz and for capacitance at 32 kHz, respectively.

### Statistical analysis

Experiments were replicated at least three times. Significance of differences was analyzed by Two-way ANOVA and Mann-Whitney U test using Graph Pad Prism 6. P-values lower than 0.05 were considered statistically significant. Immunohistochemistry scores were quantified by ImageJ. Mean score values from image analysis were analyzed using the Kruskal-Wallis test for unpaired samples to compare percentage staining between the three subject groups, and by the Mann-Whitney U-test for unpaired samples for comparison between separate groups.

## Results

### PCDH1 localizes at cell-cell contact sites in a bronchial epithelial cell line

**Fig 1:**
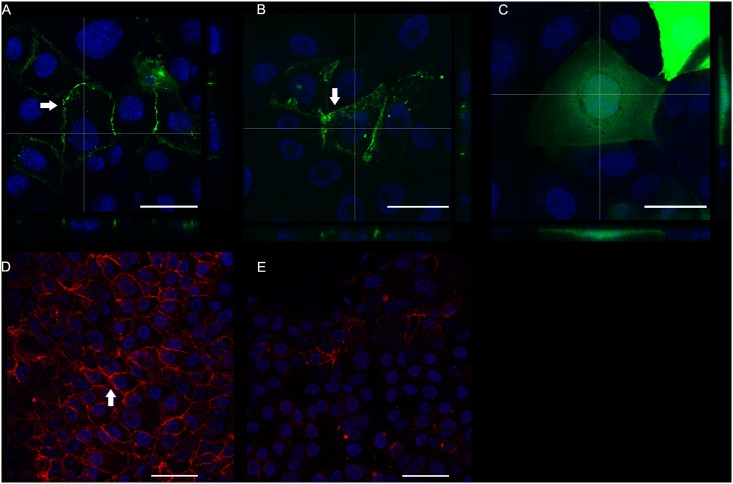
PCDH1 localizes to cell-cell contact sites in 16HBE cells. (A-C) To examine the subcellular localization of the PCDH1 isoforms (arrows), 16HBE cells were transfected with either green fluorescent protein GFP-tagged PCDH1-isoform-1 (A), PCDH1-isoform-2 (B) or control GFP-tagged cells (C). Representative images are shown from confocal z-stacks showing orthogonal cross-sections at the lines marked, and overlaid with DAPI nuclear stain. Green = PCDH1 isoform-1 or isoform-2; Blue is DAPI (nucleus). Scale bars, 30 μm. (D-E) Representative immunofluorescence overlaid images showing 16HBE cells untreated (D) and transfected with PCDH1-siRNA (E), both stained with PCDH1-EC1 antibody followed by detection with secondary goat anti-mouse Alexa Fluor 633 conjugate antibody (red) and nuclear staining with DAPI (blue). Scale bars, 50 μm.

First, we aimed to replicate the original observations from the mouse L cell fibroblast overexpression assay [18] using a bronchial epithelial cell line. To this end, fluorescently tagged PCDH1 isoforms 1 and 2 were transfected into 16HBE cells. Both GFP-tagged PCDH1 isoforms (Fig 1A–1B) localized at cell-cell contact sites but not in the GFP-transfected cells (Fig 1C). No difference in localization was observed between the two PCDH1 isoforms. Next, we determined by immunofluorescence the subcellular localization of endogenous PCDH1 protein in 16HBE cells using monoclonal antibody PCDH1-EC1, which detects the extracellular domains of both PCDH1 isoforms 1 and 2. PCDH1-specific immunoreactivity was specifically associated with the cell-cell contact sites (Fig 1D). Knockdown of PCDH1 protein decreased immunoreactivity specifically at cell-cell contact sites (Fig 1E) while the control-siRNA (negative control) did not decrease PCDH1 staining. Specificity of the PCDH1-siRNA used was confirmed by western blot [10].

### PCDH1 localized within the lateral border and basal to Adherens and Tight Junctions in differentiated primary bronchial epithelial cells

**Fig 2:**
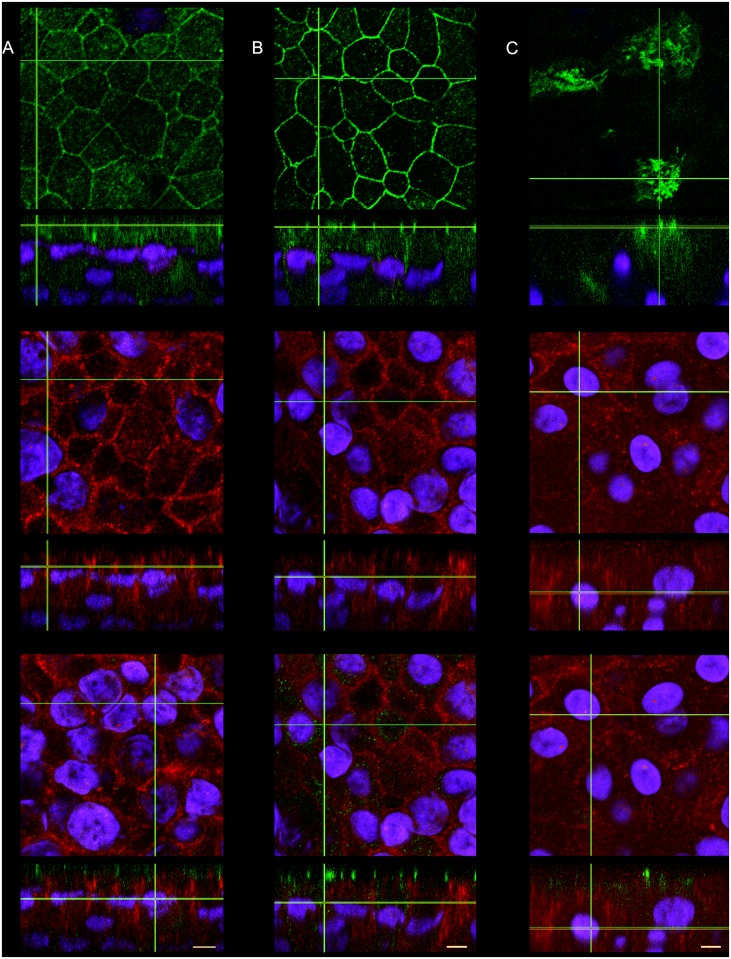
PCDH1 localized within the lateral border and basal to Adherens and Tight Junctions in differentiated primary bronchial epithelial cells. PBECs were grown for mucociliary differentiation using ALI cultures and dual-stained for with (A) E-cadherin + PCDH1, (B) Occludin + PCDH1, and (C) β-Tubulin + PCDH1. Representative immunofluorescence images from confocal z-stacks showing orthogonal image cross-sections at the lines marked overlaid with DAPI nuclear stain. Top panel images showing: (A) E-cadherin (AJ), (B) Occludin (TJ) and (C) β-Tubulin staining, all at z-stack position with strongest staining intensity. Middle panels showing: PCDH1 staining. Lower panel images: overlay of the 3 channels taken at the z-stack position with the strongest PCDH1 signal. E-cadherin, Occludin and β-Tubulin = green; PCDH1 = red and DAPI staining of the nucleus = blue. Scale bars, 5 μm.

Next, we investigated the subcellular localization of PCDH1 using differentiated PBECs derived from control subjects and cultured for 28 days under ALI conditions. This was done in relation to the classical adherens junction protein E-cadherin (AJ) and the tight junction (TJ) protein Occludin using confocal microscopy. We observed both, E-cadherin and Occludin protein located at the apico-lateral border of the membrane (Fig 2A and 2B**–Top panel**). Orthogonal cross-sections indicated that, while PCDH1 and E-cadherin localize to the cell membrane and display close proximity, they do not co-localize. Specifically, we observed that membrane staining of PCDH1 localized basal to E-cadherin within the lateral cell borders cell, with no overlap at all (Fig 2A**–Bottom panel)**. Noteworthy, PCDH1 protein localized even more basal to the Occludin signal (Fig 2B, orthogonal cross-sections). Dual-staining of PCDH1 and β-tubulin protein (as a marker of cilia) confirmed the presence of ciliated cells, but no co-localization with PCDH1 was observed (Fig 2C).

### PCDH1 expression increases during differentiation of PBECs in ALI culture

**Fig 3:**
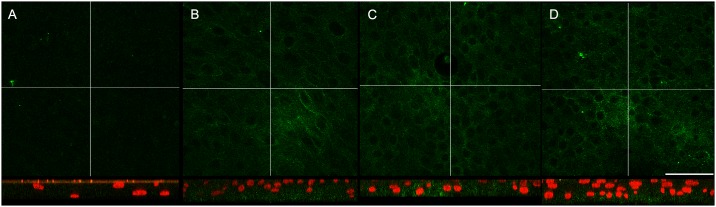
PCDH1 expression increases during differentiation of PBECs ALI cultures. PBECs cultured under ALI cultures until mucociliary differentiation and were fixed at different time points, namely day 0, 07, 14 and 28. Immunostaining was with monoclonal antibody PCDH1-EC1. Representative single channel immunofluorescence images were from confocal z-stacks showing orthogonal X-Z cross-sections at the lines marked, and overlaid with DAPI nuclear stain. Images for (A) 0, (B) 07, (C) 14 and (D) 28 days are shown. PCDH1 = green and DAPI staining of the nucleus = red. Scale bar, 50 μm.

Previously, *Koning et al*. observed a clear increase of PCDH1 mRNA and protein expression levels in PBECS on the ALI during mucociliary differentiation [6]. Here, we investigated whether the subcellular localization of PCDH1 changed during mucociliary differentiation. Therefore, we cultured PBECs at the ALI and stained for PCDH1 on days 0, 7, 14, 21 and 28, in four independent experiments (Fig 3). We observed no PCDH1 expression at day 0 of air exposure. PCDH1 staining was detected at day 7 and increased over time with the strongest signal at day 21 and 28 of ALI culture (Fig 3A–3D). It is noteworthy that orthogonal cross-sections in Fig 3 demonstrate PCDH1 immunoreactivity at cell-cell contacts with a weak signal in basal cells and the strongest signal towards the apico-lateral border of the pseudo-stratified epithelial ALI culture (S1 Video). In summary, we observed that PCDH1 localizes at epithelial cell-cell contacts and displays marked localization in the apico-lateral border in the pseudo-stratified ALI cultures with increasing expression during differentiation, and no change in subcellular distribution.

### PCDH1 expression levels and subcellular localization are similar between asthmatics and control subjects

**Fig 4:**
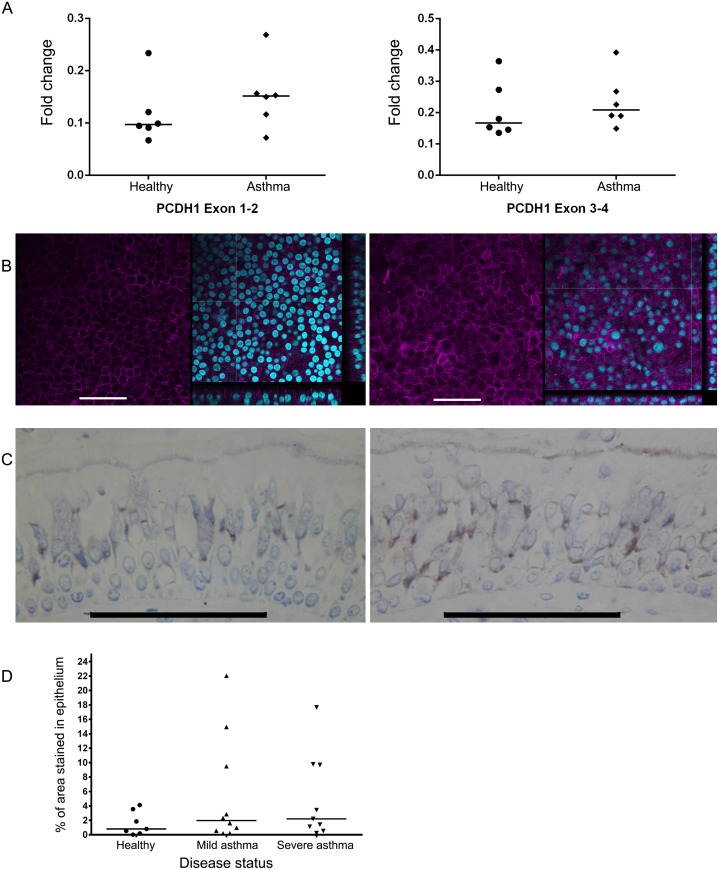
PCDH1 expression levels and subcellular localization patterns are similar between asthmatics and control subjects. A. mRNA expression levels of PCDH1 exon 1–2 (left panel) and exon 3–4 (right panel) were determined in PBECs grown until mucociliary differentiation under ALI cultures derived from control and asthmatic subjects. Independent values from 6 subjects per group and median values are shown. B. Representative immunofluorescence images of control (left panel) and asthmatic (right panel) PBECs grown until mucociliary differentiation using ALI cultures and staining with PCDH1-EC1 monoclonal antibody. PCDH1 = Magenta and DAPI staining of the nucleus = light-blue. Scale bars, 50 μm. C. Representative images of airway wall biopsies embedded in GMA from control (left panel) and asthmatic (right panel) subjects showing immunohistochemical localization of PCDH1 by use of PCDH1-EC1 antibody. Scale bar, 10 μm. D. Expression of PCDH1 in airway wall biopsies was quantified by Image J computer-aided image analysis. Dot plot showing PCDH1 protein expression in the respiratory epithelium of healthy volunteers, mild asthmatics and severe asthmatics demonstrated by percentage area staining of the epithelium. Independent values from all subjects per group and median values are shown.

Gene and protein expression levels were determined as well as the localization of PCDH1 in ALI cultured PBECs and in airway wall biopsies from asthmatics and control subjects. First, PCDH1 mRNA expression levels from differentiated PBECs were quantified by qRT-PCR. In a subsequent study, the localization pattern of PCDH1 was compared by fluorescence microscopy in ALI cultured PBECs. We observed no significant differences either in gene expression (Fig 4A), or in PCDH1 expression patterns (Fig 4B), in differentiated PBECs from asthmatic patients and control subjects. We subsequently investigated expression of PCDH1 by IHC in airway wall biopsies from asthmatics (both mild and severe) and control subjects. As shown, (Fig 4C**–Left Panel)** in control subjects staining for PCDH1 was generally weak or absent. However in some asthmatics, positive immunoreactivity was observed predominantly in the perinuclear region and localized to the baso-lateral membranes in columnar epithelial cells of the respiratory epithelium, with apical staining often associated with ciliated cells rather than goblet cells (Fig 4C**–Right Panel)**. Basal cells were negative. PCDH1 expression levels were variable between individuals, and no overall significant differences were found in PCDH1 expression between controls, mild and severe asthmatics (Fig 4D).

### PCDH1 mediates homotypic adhesion interaction

**Fig 5:**
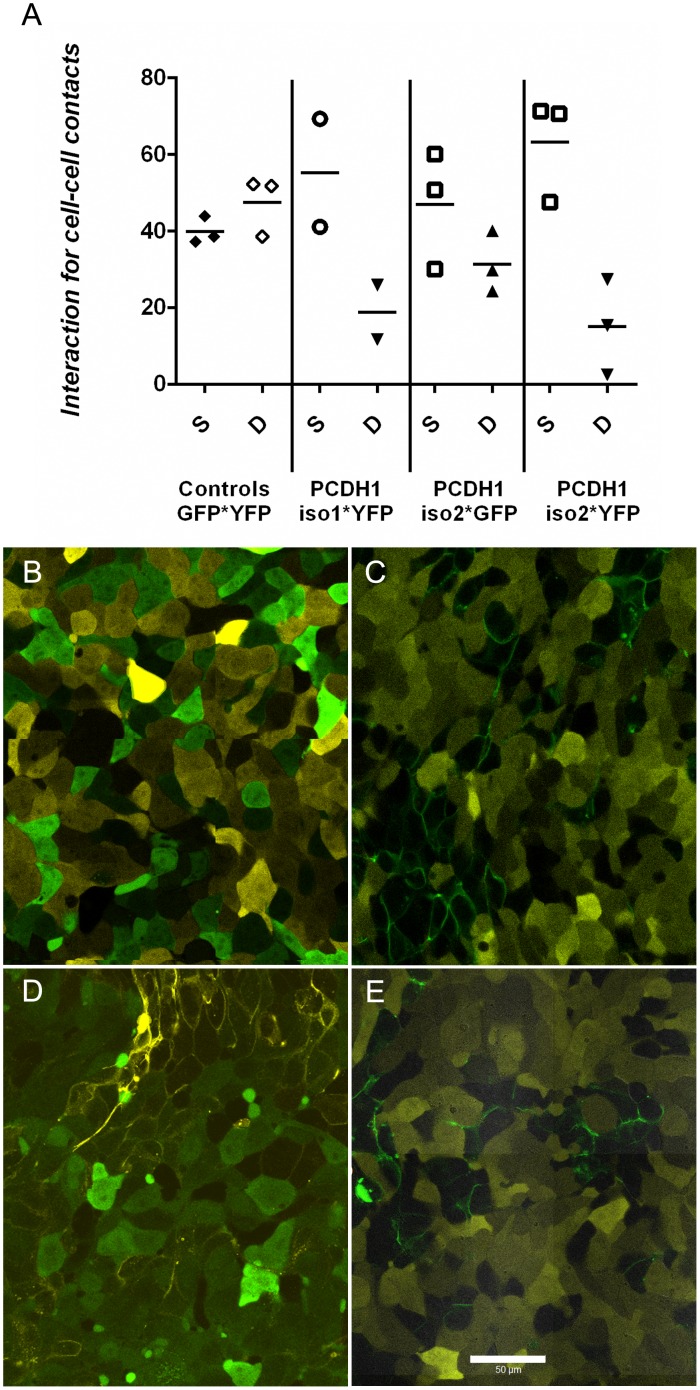
PCDH1 mediates homotypic adhesion interaction. Combination of HEK293T cells stably expressing human PCDH1-isoforms mixed and allowed to adhere in suspension for 4 hours at 37°C were plated for 16 h before imaging. (A) The Y-axis values represent the mean of 9 measurement of the fraction of cells being in contact with a “reference cell”. This one being either identical to the reference cell (SELF) or the other cell type mixed in with the reference cell (DIFFERENT). SELF (S) represents the % of all aligning cells with the reference cell which had the same color (i.e. same transfected isoform or empty vector). DIFFERENT (D) represents the % cells of all cells aligning with the reference cell which were of different color. The line represents the mean value of the three independent experiments. (B-E) Representative clones combinations images are shown. (B) GFP * YFP: both HEK293T clones as negative control. (C) GFP-tagged-PCDH isoform 1 and YFP-control cells. (D) YFP-tagged-PCDH1 isoform 2 and GFP-control cells. (E) GFP-tagged-PCDH isoform 2 and YFP-control cells. GFP = green and YFP = yellow. Scale bar, 50 μm. *Data not available for YFP-tagged-PCDH1 isoform 1 and GFP-control cells.

PCDH1 has been proposed to play a role in mediating cell sorting [18,19]. Therefore, we investigated the role of PCDH1 isoforms in establishing cell-cell contacts using cultured HEK293T cells stably overexpressing either GFP or YFP (negative controls), GFP-tagged PCDH1-isoform-1 or GFP or YFP-tagged PCDH1-isoform-2. Interaction or adjacent localization of PCDH1 isoforms strongly suggested the occurrence of homotypic interactions. As shown in Fig 5A, control HEK293T-cells transfected with either the control YFP or GFP did not show any tendency for preferential cell-cell contacts as evident from the expected 50% self and 50% different cells adhering to the reference cell. In contrast, HEK293T cells transfected with PCDH1 isoform-1 or isoform-2 showed preferential binding to cells expressing PCDH1 (Fig 5C–5E). These data support the notion that PCDH1 mediated cell-cell adhesion through homotypic interactions.

### Downregulation of PCDH1 reduces epithelial barrier function and repair

**Fig 6:**
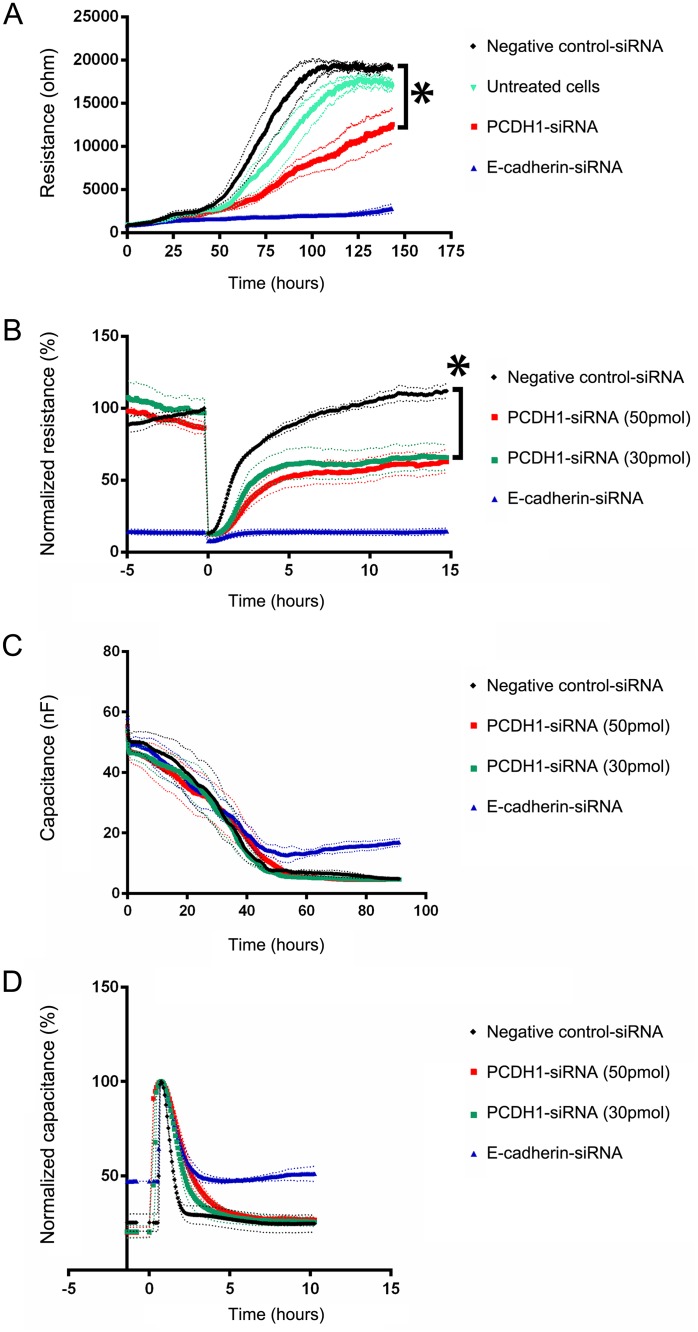
Downregulation of PCDH1 reduces epithelial barrier function and repair. A. Downregulation of endogenous expression of PCDH1 in 16HBE bronchial epithelial cells reduced and delayed build-up of electrical resistance during growth of the cells to confluence. Untreated 16HBE cells or cells previously transfected with either PCDH1-siRNA, or E-cadherin-siRNA or control-siRNA were seeded in duplicates into ECIS 8-well arrays for real-time measurement of the electrical resistance over a time course of 150 h, at 400 Hz. B. Knockdown of endogenous expression of PCDH1 in confluent 16HBE bronchial epithelial cells affected the restoration of epithelial barrier after wounding. 16HBE cells were seeded in duplicates into 8-well ECIS arrays and allowed to attach for 24 h. Then, they were transfected with either PCDH1-siRNA, E-cadherin-siRNA or control-siRNA. Upon 90 h of siRNA transfection cells were wounded by electroporation using voltage pulses of 5 V and 40 kHz for 30 sec (time is 0 h). Thereafter, electrical resistance was monitored for 17 h. C. Knockdown of endogenous expression of PCDH1 in 16HBE bronchial epithelial cells did not affect build of electrical capacitance during growth of the cells to confluence. 16HBE cells transfected with either PCDH1-siRNA, E-cadherin-siRNA or control-siRNA were seeded in duplicates into ECIS 8-well arrays for real-time measurement of the electrical capacitance over a time course of 100 h, at 32 Hz. D. Knockdown of endogenous expression of PCDH1 in confluent 16HBE bronchial epithelial cells does not affect electrical capacitance of the epithelial barrier after wounding. 16HBE cells were seeded in duplicates into 8-well ECIS arrays and allowed to attach for 24 h. Then, they were transfected with either PCDH1-siRNA, E-cadherin-siRNA or control-siRNA. Upon 90 h of siRNA transfection cells were wounded by electroporation using voltage pulses of 5 V and 40 kHz for 30 sec (time is 0 h). Thereafter, capacitance was monitored for 10 h. Data was normalized to peak capacitance after wounding. Mean values from four independent experiments are shown and error lines (dotted) indicate standard error of the mean. * p <0.001 of PCDH1-siRNA compared to control-siRNA (using two-way ANOVA).

PCDH1 is not a classical cell-cell adhesion molecule as its adhesion properties were reported to be weak [18]. Therefore, we investigated whether PCDH1 contributes to acquisition and maintenance of the bronchial epithelial barrier. First, we tested whether knockdown of PCDH1 by siRNA in 16HBE cells would affect the build-up of barrier function during growth of the cells to confluence, using an E-cadherin-siRNA as a positive control for lack of barrier formation [20]. To this end, untreated 16HBE cells or cells previously transfected with either PCDH1-specific-siRNA, E-cadherin-siRNA or negative control-siRNA were seeded on ECIS wells and build-up of electrical resistance was measured in real-time. As expected, we observed a clear increase of resistance in untreated 16HBE cells, reaching a plateau at 100 h, reflecting efficient formation of cell-cell contacts [12]. In agreement with previous data, knockdown of E-cadherin by siRNA fully abrogated build-up of resistance during the course of the experiment (≤ 125 h). Interestingly, knockdown of PCDH1 resulted in significantly delayed and reduced build-up of resistance, reaching a plateau at approximately half of the resistance of the control-siRNA treated cells (Fig 6A). In a subsequent experiment, we tested whether knockdown of PCDH1 in confluent 16HBE cells might affect the restoration of epithelial barrier after wounding [12]. Here, cells were first seeded on the ECIS wells and transfected after 24 h with either PCDH1-siRNA, E-cadherin-siRNA or negative control-siRNA during the growth curve, followed by wounding after reaching confluence. As shown in Fig 6B, under these experimental conditions, this delayed knockdown of PCDH1 did not affect the build-up of electrical resistance in the confluent monolayer of 16HBE cells. Measurements of capacitance showed no effect on cell-matrix interactions (Fig 6C) nor after wounding (Fig 6D). However, immediately after wounding, negative control-siRNA treated cells showed a rapid restoration of electrical resistance, reaching its original value within approximately 10 h after wounding. In contrast, knockdown of PCDH1 resulted in delayed repair and incomplete (50%) recovery of the barrier resistance. These data indicate that loss of PCDH1 affects epithelial barrier function, both during initial build-up and after wounding of a confluent layer of bronchial epithelial cells. After wounding, the E-cadherin knockdown, but not the PCDH1 knockdown conditions, display a residual capacitance. Taken together, these data show that knockdown of PCDH1 does not affect electrical capacitance in 16HBE cultures, indicating that knockdown of 16HBE does not have a dominant effect on cell-matrix interactions.

## Discussion

*PCDH1* gene variants are reported to be associated with BHR [1], asthma [1,3,4] and eczema [2,4]. Here, we show for the first time that PCDH1 isoform 1 and 2 localize to the cell membrane in bronchial epithelial cells, mediating homotypic interaction. PCDH1 localized basal to Adherens and Tight Junctions along the lateral border in differentiated primary bronchial epithelial cells, with no PCDH1 expression observed near the TJs or the basal bodies of the cilia. Moreover, we show that this supra-basal lateral cell membrane staining for PCDH1 is increasing during differentiation of PBECs on ALI. No differences were detected in the expression and localization patterns of PCDH1 in PBECs grown in ALI or in airway wall biopsies from asthma patients vs. control subjects. Importantly, loss of PCDH1 reduced epithelial barrier function, both during establishment of the barrier as well as during epithelial repair after damage, indicating that dysregulation of PCDH1 might contribute significantly to loss of epithelial integrity in specific subgroups of asthma patients.

Our data elucidate a dual role for PCDH1 in regulating repair responses of airway epithelial cells. We recently reported that two PCDH1 isoforms 1 and 2 interact with SMAD3 to affect TGF-beta induced responses in airway epithelial cells [10]. We now add evidence for the role of PCDH1 in epithelial barrier function and homotypic cell-cell adhesion. In addition to asthma, barrier dysfunction has also been implicated in the pathogenesis of other atopic diseases, such as eczema. Co-morbidity studies in asthma, eczema and rhinitis suggest that part of the overlap in allergic diseases may be due to non-IgE mediated mechanisms [21], and we propose that loss of epithelial integrity may be one of these mechanisms involved. This hypothesis is supported by genetic findings on the *Fillagrin* encoding gene *FLG*, where loss-of-function mutations were strongly associated with eczema, and also with asthma in patients with eczema [22]. Recently, the gene encoding cadherin-related family member 3 (*CDHR3*), another putative cell-cell adhesion molecule, was found to be associated with childhood asthma in a genome wide association study, strengthening the genetic evidence that loss of epithelial barrier function may be one of the fundamental mechanisms in asthma development [23]. Recently, it was shown that *CDHR3* is involved in binding of Rhinovirus C and mediated entering of rhinovirus C into the host cells [24], providing a model of epithelial susceptibility for viral infections.

The airway epithelium represents the first line of defense of the respiratory tract. Through intercellular adhesion complexes and the mucociliary layer, it provides a size and ion-selective physical barrier to environmental insults [25,26]. In healthy airway epithelium, TJs and AJs maintain the physical barrier as well as the apico-basal polarity of this highly organized epithelium. The integrity of the airway epithelium in asthmatics is compromised due to a weakened barrier function [7,27], increased permeability to allergens, detachment of ciliated cells and loss of cell-cell junctional molecules such as ZO-1 and E-cadherin [28,29]. As a consequence, the asthmatic epithelium can respond to environmental insults by producing increased amounts of pro-inflammatory cytokines, which will contribute to a chronically inflamed phenotype.

Could dysfunction of PCDH1 compromise the airway epithelial barrier of the asthmatic epithelium? We provide some evidence that this might indeed be the case, although we did so far not identify the exact subgroup of asthma patients in which this can be conclusively shown. A role of PCDH1 in epithelial barrier function [9] is strongly suggested by the expression of *PCDH1* at the cell-cell contact sites of airway epithelial cells localizing baso-laterally to the AJ, and its increased expression during mucociliary differentiation of healthy bronchial epithelial cells [6], for which adhesion function is an important factor. As, we found that a decreased of PCDH1 expression results in epithelial barrier dysfunction and an impaired repair response. The divergent effects of knockdown of PCDH1 on electrical resistance versus capacitance, with no effect of PCDH1 knockdown on electrical capacitance during 16HBE growth and during wound repair seem to indicate that the effects of PCDH1 are selective for epithelial barrier formation due to junctional complex formation at cell-cell contacts, while cell-matrix adhesion is not affected by PCDH1 loss. Therefore, loss of PCDH1 functionality may indeed explain some of the phenotypes seen in the airway epithelium of asthmatics. Nevertheless, it remains unknown how PCDH1 would interact with E-cadherin or other molecules of the epithelial adhesion complex to mediate its effect on epithelial barrier function, how reduced barrier function due to loss of PCDH1 would affect permissiveness of the epithelium to large molecules such as FITC-Dextran, and in what specific subgroup of asthma patients PCDH1 would show this effect. Further functional studies, that include analysis of PCDH1 in subcellular fractions as well as identification of protein interaction partners, are needed to address these questions.

We tested whether PCDH1 was differentially expressed between asthma and control subjects by three approaches, using two sources of primary material. We did not detect differences in gene expression, protein levels or localization patterns of PCDH1 in PBECs grown at ALI conditions using an antibody that detects the extracellular portion of PCDH1 isoform 1 and 2, but not isoform 3. However, this study might have been underpowered (n = 4) to detect differences at the group level, especially since we did not stratify for *PCDH1* genotype in our analyses. The immunohistochemically staining of airway tissues was better powered, but here we mainly observed large-within-group variability, without significant differences in PCDH1 protein levels or cellular distribution patterns between groups. Using immunohistochemistry, *Kuzo et al*. 2015, observed no differences in the distribution of PCDH1 in the nasal tissues of Japanese patients with chronic rhinosinusitis compared to the airway of asthmatic patients with similar histological appearance [9]. Since we analyzed PCDH1 localization and expression using an antibody directed against the extracellular domain, we were unable to differentiate between PCDH1 isoforms that only differ in the intracellular protein domains. Therefore, we cannot draw any conclusions regarding the regulation of PCDH1 isoforms during epithelial differentiation on ALI and between asthma and control subjects. Moreover, in this analysis, a role for the *PCDH1* genotype, including a potential effect of smoking on gene expression, was not investigated [30]. Finally, *Kuzo et al*. suggested that PCDH1 is upregulated by corticosteroids, which may in part explain the increased expression we observed in asthmatics [9]. Further studies comparing PCDH1 levels in airway tissue between asthmatic individuals using ICS and those that are not using ICS might shed light on this important issue. We conclude that while decreased PCDH1 might contribute to airway epithelial dysfunction in asthma, altered PCDH1 expression levels do not seem to be a shared mechanism in all asthma patients, and further research will need to address whether loss of PCDH1 contributes to airway epithelial barrier dysfunction in those asthma patients carrying the asthma-susceptible *PCDH1* allele.

In order to further interpret our findings, several strengths and limitations need to be considered. First, to our knowledge we are the first to identify the localization of PCDH1 isoforms in bronchial epithelial cell lines using an overexpression system as well as detection of endogenous expression in primary airway epithelium. Moreover, we confirmed the specificity of PCDH1 staining by PCDH1 knockdown and the different approaches used yielded highly consistent results. A drawback from our study is that we could not perform these studies in asthmatic subjects and controls stratified for the *PCDH1* asthma susceptibility genotypes, since there was no informed consent to genotype these subjects. The question how *PCDH1* gene variants regulate PCDH1 expression or function is therefore still open. Moreover, it is well known that airway epithelial cells of asthma patients display an immature phenotype characterized by increased expression of basal cell markers [31]. If a reduced capacity of airway epithelial cells to differentiate is a basic defect that partly determines asthma susceptibility, the use of well differentiated airway epithelial cells may have introduced a bias to our studies. The same holds true for the analysis of airway wall biopsies. Finally, differences in the localization of specific *PCDH1* isoforms or changes in the balance between isoforms 1 and 2 could not be determined as the antibodies specific for PCDH1 isoforms, generated by us in the context of previous studies [6], did not meet the validation criteria for specificity and reproducibility of immunostainings. We suggest that further work should be performed to identify the relative expression and specific roles of PCDH1 isoforms.

In conclusion, we provide strong evidence that PCDH1 may function as a cell-cell adhesion molecule involved in epithelial repair. We showed that PCDH1 localizes to cell-cell contact sites apicolaterally in differentiated airway epithelium and that PCDH1 expression becomes stronger toward the most differentiated cells. Importantly, we provide evidence that PCDH1 function is relevant for maintaining epithelial barrier and that loss of functional PCDH1 represents a potential mechanism for development of asthma and BHR. This may lead to novel therapeutic approaches aiming to improve or reconstitute epithelial barrier function in asthma.

## Supporting Information

S1 Fig PCDH1 localized within the lateral border and basal to Adherens and Tight Junctions in differentiated primary bronchial epithelial cells. E-cadherin, Occludin and β-Tubulin = yellow; PCDH1 = magenta and DAPI staining of the nucleus = light-blue. Scale bars, 5 μm.
(TIF)

S1 Video PCDH1 localization at cell-cell contacts with strongest signal towards the apicolateral borders of the pseudo-stratified epithelial ALI culture. Representative PBECs cultured at ALI culture until mucociliary differentiation were fixed at day 28, stained with monoclonal antibody PCDH1-EC1 and labeled with Alexa Fluor^®^ 488 conjugated secondary antibody. Overlay confocal images of PCDH1 (green) and DAPI staining of the nucleus (red) were taken using a laser-scanning confocal microscope. Frames were taken every 0.5 μm, from basal to apical. Scale bar, 50 μm.
(7Z) Click here for additional data file.

## References

[1] Koppelman GH, Meyers DA, Howard TD, Zheng SL, Hawkins GA, Ampleford EJ, et al Identification of PCDH1 as a novel susceptibility gene for bronchial hyperresponsiveness. Am J Respir Crit Care Med. 2009;180: 929–935. 10.1164/rccm.200810-1621OC 19729670 PMC2778155

[2] Koning H, Postma DS, Brunekreef B, Duiverman EJ, Smit HA, Thijs C, et al Protocadherin-1 polymorphisms are associated with eczema in two Dutch birth cohorts. Pediatr Allergy Immunol. 2012;23: 270–277. 10.1111/j.1399-3038.2011.01201.x 21929597

[3] Toncheva AA, Suttner K, Michel S, Klopp N, Illig T, Balschun T, et al Genetic variants in Protocadherin-1, bronchial hyper-responsiveness, and asthma subphenotypes in German children. Pediatr Allergy Immunol. 2012;23: 636–641. 10.1111/j.1399-3038.2012.01334.x 23050600

[4] Mortensen LJ, Kreiner-Moller E, Hakonarson H, Bonnelykke K, Bisgaard H. The PCDH1 gene and asthma in early childhood. Eur Respir J. 2014;43: 792–800. 10.1183/09031936.00021613 23988763

[5] Faura Tellez G, Nawijn MC, Koppelman GH. Protocadherin-1: Epithelial barrier dysfunction in asthma and eczema. Eur Respir J. 2014;43: 671–674. 10.1183/09031936.00179713 24585862

[6] Koning H, Sayers I, Stewart CE, de Jong D, Ten Hacken NH, Postma DS, et al Characterization of protocadherin-1 expression in primary bronchial epithelial cells: association with epithelial cell differentiation. FASEB J. 2012;26: 439–448. 10.1096/fj.11-185207 21982948

[7] Xiao C, Puddicombe SM, Field S, Haywood J, Broughton-Head V, Puxeddu I, et al Defective epithelial barrier function in asthma. J Allergy Clin Immunol. 2011;128: 549–56.e1–12. 10.1016/j.jaci.2011.05.038 21752437

[8] Heijink IH, Nawijn MC, Hackett TL. Airway epithelial barrier function regulates the pathogenesis of allergic asthma. Clin Exp Allergy. 2014;44: 620–630. 10.1111/cea.12296 24612268

[9] Kozu Y, Gon Y, Maruoka S, Kazumichi K, Sekiyama A, Kishi H, et al Protocadherin-1 is a glucocorticoid-responsive critical regulator of airway epithelial barrier function. BMC Pulm Med. 2015;15: 80-015-0078-z. 10.1186/s12890-015-0078-z 26227965 PMC4521469

[10] Faura Tellez G, Vandepoele K, Brouwer U, Koning H, Elderman RM, Hackett TL, et al Protocadherin-1 binds to SMAD3 and suppresses TGF-beta1-induced gene transcription. Am J Physiol Lung Cell Mol Physiol. 2015;309: L725–35. 10.1152/ajplung.00346.2014 26209277 PMC4593836

[11] Cozens AL, Yezzi MJ, Yamaya M, Steiger D, Wagner JA, Garber SS, et al A transformed human epithelial cell line that retains tight junctions post crisis. In Vitro Cell Dev Biol. 1992;28A: 735–744. 10.1007/BF02631062 1282914

[12] Heijink IH, Brandenburg SM, Noordhoek JA, Postma DS, Slebos DJ, van Oosterhout AJ. Characterisation of cell adhesion in airway epithelial cell types using electric cell-substrate impedance sensing. Eur Respir J. 2010;35: 894–903. 10.1183/09031936.00065809 19741028

[13] You Y, Richer EJ, Huang T, Brody SL. Growth and differentiation of mouse tracheal epithelial cells: selection of a proliferative population. Am J Physiol Lung Cell Mol Physiol. 2002;283: L1315–21. 10.1152/ajplung.00169.2002 12388377

[14] [Anonymous]. Workshop summary and guidelines: investigative use of bronchoscopy, lavage, and bronchial biopsies in asthma and other airway diseases. J Allergy Clin Immunol. 1991;88: 808–814. 1955640 10.1016/0091-6749(91)90189-u

[15] Bucchieri F, Puddicombe SM, Lordan JL, Richter A, Buchanan D, Wilson SJ, et al Asthmatic bronchial epithelium is more susceptible to oxidant-induced apoptosis. Am J Respir Cell Mol Biol. 2002;27: 179–185. 10.1165/ajrcmb.27.2.4699 12151309

[16] Britten KM, Howarth PH, Roche WR. Immunohistochemistry on resin sections: a comparison of resin embedding techniques for small mucosal biopsies. Biotech Histochem. 1993;68: 271–280. 10.3109/10520299309105629 8268322

[17] Schindelin J, Arganda-Carreras I, Frise E, Kaynig V, Longair M, Pietzsch T, et al Fiji: an open-source platform for biological-image analysis. Nat Methods. 2012;9: 676–682. 10.1038/nmeth.2019 22743772 PMC3855844

[18] Sano K, Tanihara H, Heimark RL, Obata S, Davidson M, St John T, et al Protocadherins: a large family of cadherin-related molecules in central nervous system. EMBO J. 1993;12: 2249–2256. 8508762 10.1002/j.1460-2075.1993.tb05878.x PMC413453

[19] Saburi S, Hester I, Goodrich L, McNeill H. Functional interactions between Fat family cadherins in tissue morphogenesis and planar polarity. Development. 2012;139: 1806–1820. 10.1242/dev.077461 22510986 PMC3328180

[20] Heijink IH, Kies PM, Kauffman HF, Postma DS, van Oosterhout AJ, Vellenga E. Down-regulation of E-cadherin in human bronchial epithelial cells leads to epidermal growth factor receptor-dependent Th2 cell-promoting activity. J Immunol. 2007;178: 7678–7685. 10.4049/jimmunol.178.12.7678 17548604

[21] Pinart M, Benet M, Annesi-Maesano I, von Berg A, Berdel D, Carlsen KC, et al Comorbidity of eczema, rhinitis, and asthma in IgE-sensitised and non-IgE-sensitised children in MeDALL: a population-based cohort study. Lancet Respir Med. 2014;2: 131–140. 10.1016/S2213-2600(13)70277-7 24503268

[22] Rodriguez E, Baurecht H, Herberich E, Wagenpfeil S, Brown SJ, Cordell HJ, et al Meta-analysis of filaggrin polymorphisms in eczema and asthma: robust risk factors in atopic disease. J Allergy Clin Immunol. 2009;123: 1361–70.e7 10.1016/j.jaci.2009.03.036 19501237

[23] Bonnelykke K, Sleiman P, Nielsen K, Kreiner-Moller E, Mercader JM, Belgrave D, et al A genome-wide association study identifies CDHR3 as a susceptibility locus for early childhood asthma with severe exacerbations. Nat Genet. 2014;46: 51–55. 10.1038/ng.2830 24241537

[24] Bochkov YA, Watters K, Ashraf S, Griggs TF, Devries MK, Jackson DJ, et al Cadherin-related family member 3, a childhood asthma susceptibility gene product, mediates rhinovirus C binding and replication. Proc Natl Acad Sci U S A. 2015;112: 5485–5490. 10.1073/pnas.1421178112 25848009 PMC4418890

[25] Vareille M, Kieninger E, Edwards MR, Regamey N. The airway epithelium: soldier in the fight against respiratory viruses. Clin Microbiol Rev. 2011;24: 210–229. 10.1128/CMR.00014-10 21233513 PMC3021210

[26] Knight DA, Holgate ST. The airway epithelium: structural and functional properties in health and disease. Respirology. 2003;8: 432–446. 10.1046/j.1440-1843.2003.00493.x 14708552

[27] Knight DA, Stick SM, Hackett TL. Defective function at the epithelial junction: a novel therapeutic frontier in asthma? J Allergy Clin Immunol. 2011;128: 557–558. 10.1016/j.jaci.2011.07.031 21878242

[28] de Boer WI, Sharma HS, Baelemans SM, Hoogsteden HC, Lambrecht BN, Braunstahl GJ. Altered expression of epithelial junctional proteins in atopic asthma: possible role in inflammation. Can J Physiol Pharmacol. 2008;86: 105–112. 10.1139/y08-004 18418437

[29] Lambrecht BN, Hammad H. The airway epithelium in asthma. Nat Med. 2012;18: 684–692. 10.1038/nm.2737 22561832

[30] Koning H, van Oosterhout AJ, Brouwer U, den Boef LE, Gras R, Reinders-Luinge M, et al Mouse protocadherin-1 gene expression is regulated by cigarette smoke exposure in vivo. PLoS One. 2014;9: e98197 10.1371/journal.pone.0098197 24992194 PMC4081120

[31] Roth HM, Wadsworth SJ, Kahn M, Knight DA. The airway epithelium in asthma: developmental issues that scar the airways for life? Pulm Pharmacol Ther. 2012;25: 420–426. 10.1016/j.pupt.2012.09.004 23022283

