# Supplementary figures and images for: Protocadherin-1 Localization and Cell-Adhesion Function in Airway Epithelial Cells in Asthma
Source: PLoS One. 2016 Oct 4;11(10):e0163967. doi: 10.1371/journal.pone.0163967 (PMC5049773; doi:10.1371/journal.pone.0163967)

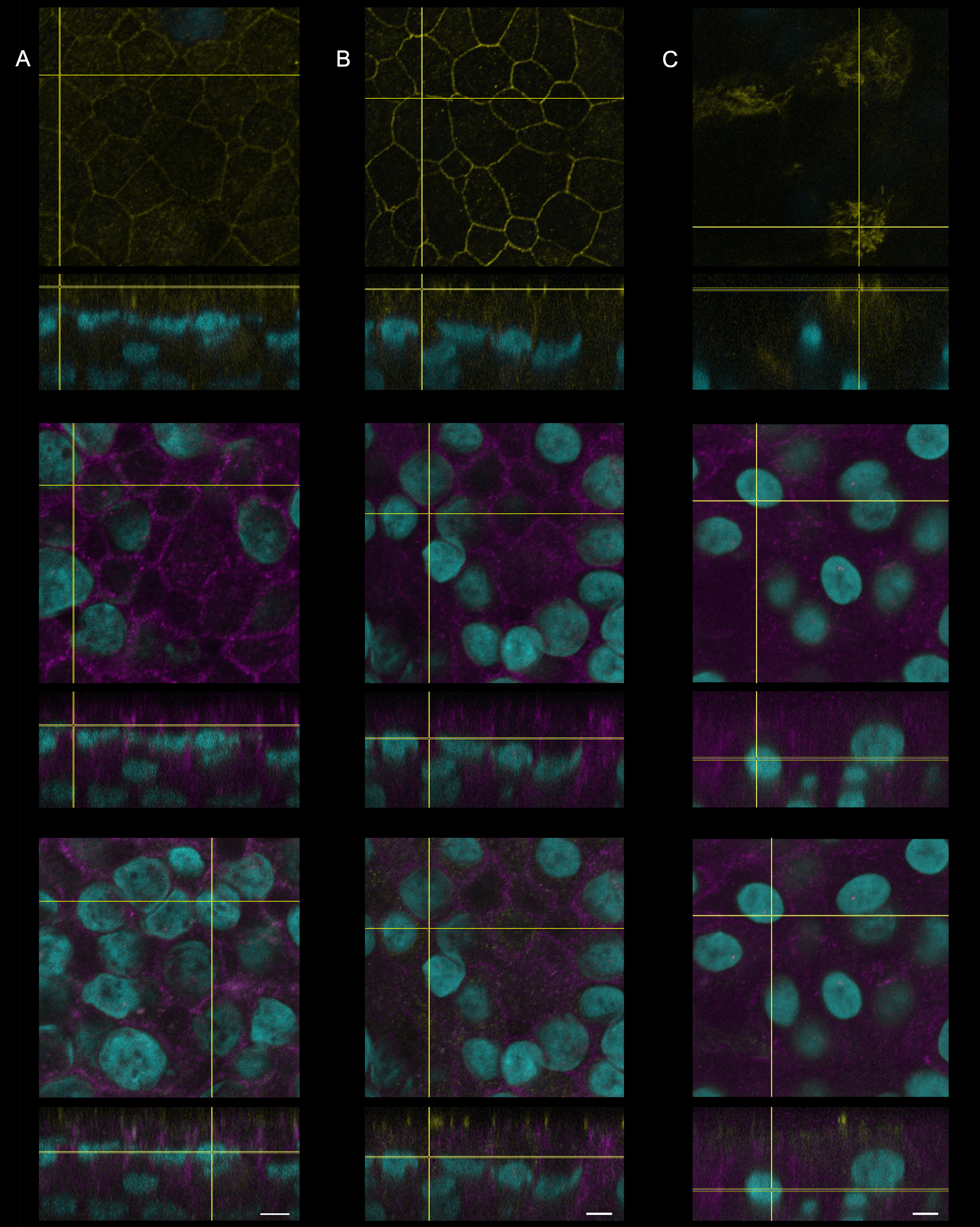

Supplement: S1 Fig — E-cadherin, Occludin and β-Tubulin = yellow; PCDH1 = magenta and DAPI staining of the nucleus = light-blue. Scale bars, 5 μm. (TIF) [file pone.0163967.s001.tif]
